# Health, subjective financial situation and well-being: a longitudinal observational study

**DOI:** 10.1186/s12955-020-01456-3

**Published:** 2020-06-26

**Authors:** Paul Downward, Simona Rasciute, Harish Kumar

**Affiliations:** 1 School of Sport, Exercise and Health Sciences, Loughborough University, Ashby Road, Loughborough, LE11 3TU UK; 2 School of Business and Economics, Loughborough University, Ashby Road, Loughborough, LE11 3TU UK

**Keywords:** Subjective financial situation, General health, Mental health, Gender

## Abstract

**Background:**

An individual’s financial situation is a key contributor to their overall well-being. Existing research has examined the direct economic consequences of changes in health upon out-of-pocket healthcare expenditure, participation in the labour force and potential earnings. There is also research exploring an individual’s concern about their subjective financial situation regardless of the level of their income or work status on their health. In contrast, this paper conducts a causal analysis of the effects of general and mental health on an individual’s subjective evaluation of their financial situation controlling for their work status and income. This is of importance because current health policy in the United Kingdom (UK) stresses the role of health as an asset which can mediate the wider flourishing of individuals. Moreover, subjective financial situation comprises a key component of well-being now being measured and sought in social welfare policy.

**Methods:**

Fixed effects instrumental variable panel data regression analysis is applied to 25 years of longitudinal data, from 1991, drawn from the harmonised British Household Panel Survey (BHPS) and Understanding Society Survey (USS).

**Results:**

Improved general health and reduced mental illness both improve the subjective financial situation of males and females. However, these affects diminish across older cohorts of males and females.

**Conclusions:**

Investing in and improving general and mental health can improve the subjective financial situation and hence well-being of individuals. The targeting of health also needs to take account of an individuals’ life-stage.

## Background

There is extensive evidence on the socio-determinants of health (SDH) [1]. It is argued that the SDH produce health disparities because, depending on the context, a lack of social and economic resources can reduce access to health care, only permit access to lower quality health care, or encourage engagement in more harmful lifestyles [2, 3].

The association between access to health and objective economic inequality has been investigated broadly through a focus on three issues: access to out-of-pocket healthcare expenses; restricted participation in the labour market; and, reduced potential for earnings. The relative importance of these relationships, however, is country-specific, depending on the healthcare system.

In the United States (US) and Australia individuals with health conditions face out-of-pocket healthcare expenditure. The expenses are found to be associated with an increase in the total number of chronic conditions of individuals [4–7], but also specific conditions such as chronic obstructive pulmonary disease in Australia [8], cancer in the US [9], high blood pressure, diabetes or depression in Australia [6]. Moreover, poor health is also associated with other costs of illness, such as transportation and configuration of home care environments, in the US [2].

Depression or mood affective disorders, diseases of the respiratory system, diseases of the circulatory system (other than hypertension), heart diseases, and mental and behavioural disorders are also found to be associated with being out of the labour force in Australia [10, 11]. Back problems and arthritis are associated with early retirement in Australia [11]. Being out of the labour force is associated with musculoskeletal conditions in the US [12], multiple sclerosis in Sweden [13] and bipolar disorder in the US [14]. An association between undergoing active cancer care and less likelihood of being employed full-time is also found in the US [15].

Poorer health is also found to be associated with the lower potential to earn income. For example, individuals suffering from multiple health conditions have been found to be associated with lower incomes and possession of less assets, a higher propensity to hold consumer debt, higher risk of cash flow difficulties, dissaving to meet day-to-day living expenses and exclusion from financial providers in Australia [16] and the US [17]. Moreover, episodes of poor health earlier in life through reduced earnings are shown to be negatively associated with the level of pension and social security income during retirement. Finally new health problems, especially severe ones, may lead to people revising their expectations of how long they are going to live, and consequently may encourage a move towards present consumption [2].

The above literature focusses on actual or objective economic inequalities. However, research also recognises that subjective financial well-being is distinct from, for example, objective measures of income in connection with health and well-being [18]. This is because the same level of income may be sufficient for one person’s needs but not another [19]. In part this will be because individuals’ sense of social comparison and expectation underpin differences in the perceived adequacy of income [20], as identified in Mexico, and this in turn is likely to vary across contexts and cultures [21], as for example, shown across Europe.

A focus on subjective financial situation, as opposed to just focussing on income and employment prospects, also takes into account an individual’s capacity to participate in wider social and cultural activities as these may incur costs [22]. Moreover, anxieties that individuals feel when contemplating their financial futures may also play a fundamental role in influencing their well-being and quality of life, as individuals might be affected by the prospect of loss and the fear of experiencing financial hardship, regardless of their current stated economic circumstances. Thus, an individual’s sensitivity to risk is partially, at least, independent of their current income level, as demonstrated in Australia [23].

The relationship between health and subjective financial situation in the United Kingdom (UK) is thus explored in this paper. The context for the research is that there is growing policy development in the UK recognising that health is an asset that allows for the wider flourishing of individuals [24, 25]. Public policy in the UK also emphasises a need to promote the well-being of individuals, of which financial security is a key component [26, 27]. Consequently, this paper conducts a causal analysis of the effects of health on the subjective financial situation of individuals, which is a major contributor to their well-being, controlling for their income, work status and other confounders.

## Methods

An instrumental variable fixed effects panel-data analysis is applied to 25 years of longitudinal data, which comprises a combination of 18 years of British Household Panel Survey (BHPS) data from 1991 to 2008, and 7 years of Understanding Society: The UK Household Longitudinal Study survey (USS) data from 2009 to 2015–16. The newer USS dataset has incorporated the older BHPS but also expanded its coverage.

Table 1 identifies and labels the variables that are employed in the analysis. The key confounding variables include: The age of individuals in years as well as age cohorts to which individuals belong, their gender; their education level, measured as having a university degree or not; household composition measured in terms of having a partner or not, and the number of children of varying ages; work status, measured in terms of employment type, being unemployed, as well as being retired, or on maternity leave, caring for the family or being on long-term sick leave (compared to being on a government training scheme or other status); and, current income. This is the level of real gross monthly household income. It is not equivalised but, as noted above, the analysis controls for the presence of a partner and number of children in the household. Work status and income are important as they control for access to the current objective financial and economic resources of individuals.

**Table 1:** Variable Descriptions and Characteristics

		GHealth		GHQ12	
Variable	Description	Mean	Std dev.	Mean	Std. Dev.
Finnow	Subjective Financial situation (1-finding it very difficult to 5- living comfortably)	3.882	0.992	3.884	0.990
General Health	General Health scale (1 - very poor to 4 - Excellent)	2.879	0.812		
GHQ12	GHQ12 Likert scale (0 to 36)			11.104	5.445
Age	Age in years	47.688	18.075	47.437	17.961
Age0	Aged between 16 and 29 years	0.186	0.389	0.187	0.390
Age1	Aged between 30 and 64 years	0.534	0.499	0.538	0.498
Age2	Aged 65 years and above	0.280	0.449	0.274	0.446
BHPS	Data from the BHPS survey compared to the USS	0.513	0.500	0.531	0.499
Sex	Gender (1 - male, 0 female)	0.445	0.497	0.445	0.497
Couple	Married or a couple versus other marital status (1- yes, 0- no)	0.551	0.497	0.554	0.497
Higher Education	Has a degree or equivalent (1- yes, 0- no)	0.185	0.388	0.183	0.387
Child 0–2	Number of children aged 0 to 2 years	0.080	0.294	0.081	0.294
Child 3–4	Number of children aged 3 to 4 years	0.068	0.264	0.069	0.265
Child 5–11	Number of children aged 5 to 11 years	0.250	0.590	0.252	0.592
Child 12–15	Number of children aged 12 to 15 years	0.159	0.434	0.160	0.435
Real Family Income	Real gross monthly household income (£)	1677.714	1612.132	1676.393	1608.585
Self employed	Self employed (1- yes, 0- no)	0.073	0.261	0.073	0.260
Employed	Employed full time (1- yes, 0- no)	0.500	0.500	0.506	0.500
Unemployed	Unemployed (1- yes, 0- no)	0.038	0.191	0.037	0.190
Retired	Retired (1- yes, 0- no)	0.229	0.420	0.225	0.418
Maternity leave	On maternity leave (1- yes, 0- no)	0.005	0.069	0.004	0.069
Family care	Caring for the family (1- yes, 0- no)	0.065	0.246	0.065	0.246
Full-time study	In full-time study (1- yes, 0- no)	0.045	0.208	0.045	0.207
Long-term sick	On long-term sick leave (1- yes, 0- no)	0.037	0.189	0.036	0.187
England	Respondent is from England (1- yes, 0- no)	0.694	0.460	0.704	0.456
Scotland	Respondent is from Scotland (1- yes, 0- no)	0.122	0.328	0.125	0.330
Wales	Respondent is from Wales (1- yes, 0- no)	0.102	0.303	0.104	0.305
n		329,282		338,628	

The key outcome variable is the subjective view of an individual’s financial situation, 'Finnow'. It is measured by the question “How well would you say you yourself are managing financially these days?” Reponses are coded with a five-point Likert scale with the following dimensions: ‘finding it very difficult’, ‘finding it quite difficult’, ‘just about getting by’, ‘doing alright’ and ‘living comfortably’.

Two measures of health are used in the study. The first is a general health variable, measured on a five-point Likert scale. It takes into account an individual’s subjective view of their overall health, and it has been often identified with physical health [28, 29]. However, the response categories across BHPS and USS datasets are different. In the BHPS general health has the following categories: excellent, good, fair, poor and very poor. In the USS the categories are: excellent, very good, good, fair and poor. A synthesised measure was created based on the sample proportions for each category with the following four categories derived: excellent, good, fair, very poor. This new synthesised variable combined the ‘very poor’ and ‘poor’ scales in BHPS and the ‘very good’ and ‘good’ scales of USS. The second health measure is the 12 item General Health Question (GHQ12) which broadly measures mental health [30]. The Likert version is adopted in this study [31–33].

Because causal insights are sought, instrumental variables are employed in the analysis. The lagged value of health as well as dummy variables for England, Scotland and Wales compared to Northern Ireland and the Channel Isles are used as instruments. The former measures the health status that has occurred prior to the measurement of the subjective financial situation. The country dummies are assumed to capture the variation in supply of health at the country level. Following the devolution of governments in the countries of Scotland, Wales and Northern Ireland in 1999, the systems of governance across the countries have evolved in a distinct way [34].

The fixed effects panel-data estimator controls for unobserved individual characteristics, which are assumed to be constant over time, and may determine both health status and financial situation. A fixed effect was also used to control for the different surveys that comprised the combined data and, finally, robust variance-covariance matrix estimates are used to draw inferences to control for heteroscedasticity in the cross-sectional dimension [35]. This will improve the overall precision of estimates and, along with the fixed effect attenuate the effects of differences in the measurement of subjective general health.

In this study, the relevance and validity of the instruments is assessed through two tests [36]. F-tests to assess the joint relevance of the instruments in a regression of the two measures of health on the instruments and other confounding variables. The Hansen-Sargan test is performed to establish if the instrumental variables are correlated with the unobserved errors in the regressions of subjective financial situation on the health measures and other confounding variables. The tests for the relevance and validity of the instruments are reported at the bottom of Tables 2 and 3 which present the regression results. The results of the F-test and Sargan-Hansen test support the relevance and validity of the instruments, respectively. Consequently, causality for the impact of health on financial situation can be inferred.

**Table 2:** Instrumental Variable Fixed Effect Panel Estimates

	General Health All	General Health Male	General Health Female	GHQ12 All	GHQ12 Male	GHQ Female
General Health	0.110^***^	0.148^***^	0.0839^**^			
	(3.63)	(3.22)	(2.09)			
GHQ12				−0.167^***^	−0.168^***^	−0.166^***^
				(−13.06)	(−10.62)	(−8.39)
Age	0.0197^***^	0.0185^***^	0.0209^***^	0.0239^***^	0.0228^***^	0.0248^***^
	(24.94)	(15.31)	(20.01)	(26.61)	(17.49)	(20.21)
BHPS	0.225^***^	0.194^***^	0.248^***^	0.267^***^	0.222^***^	0.300^***^
	(25.04)	(15.05)	(19.95)	(23.49)	(14.01)	(18.51)
Couple	0.0789^***^	0.0146	0.127^***^	0.0664^***^	0.0286^*^	0.0964^***^
	(7.99)	(1.03)	(9.47)	(5.60)	(1.71)	(5.72)
Higher education	−0.0498^**^	− 0.0390	−0.0577^**^	− 0.0395^*^	− 0.0121	− 0.0596^*^
	(−2.34)	(−1.21)	(−2.06)	(−1.66)	(− 0.35)	(− 1.85)
Child 0–2	− 0.0607^***^	− 0.0805^***^	− 0.0422^***^	− 0.0459^***^	− 0.0637^***^	−0.0297^**^
	(−8.45)	(−7.90)	(− 4.19)	(− 5.13)	(−5.15)	(− 2.33)
Child 3–4	− 0.0565^***^	− 0.0530^***^	− 0.0545^***^	−0.0504^***^	− 0.0607^***^	−0.0405^***^
	(− 7.78)	(−4.97)	(− 5.51)	(−5.70)	(− 4.81)	(−3.27)
Child 5–11	− 0.0185^***^	− 0.0247^***^	− 0.0103	−0.0138^**^	− 0.00536	−0.0174^**^
	(−3.68)	(−3.43)	(− 1.49)	(− 2.37)	(− 0.63)	(− 2.16)
Child 12–15	− 0.0286^***^	− 0.0342^***^	−0.0218^***^	− 0.00815	−0.0161	− 0.00132
	(−5.12)	(− 4.11)	(− 2.92)	(− 1.18)	(− 1.62)	(− 0.14)
Real Family Income	0.0000496^***^	0.0000457^***^	0.0000570^***^	0.0000521^***^	0.0000447^***^	0.0000644^***^
	(27.85)	(20.95)	(18.63)	(24.39)	(17.47)	(16.68)
Self Employed	0.191^***^	0.194^***^	0.187^***^	0.150^***^	0.152^***^	0.141^***^
	(7.85)	(5.10)	(5.78)	(4.88)	(3.39)	(3.26)
Employed	0.222^***^	0.236^***^	0.212^***^	0.191^***^	0.194^***^	0.189^***^
	(9.91)	(6.47)	(7.51)	(6.58)	(4.46)	(4.84)
Unemployed	−0.277^***^	− 0.352^***^	− 0.198^***^	− 0.00549	− 0.0728	0.0598
	(−11.49)	(−9.27)	(− 6.37)	(− 0.15)	(− 1.40)	(1.09)
Retired	0.159^***^	0.165^***^	0.153^***^	0.0909^***^	0.0665	0.107^***^
	(6.80)	(4.38)	(5.15)	(2.97)	(1.43)	(2.63)
Maternity leave	0.224^***^		0.204^***^	0.134^***^		0.120^**^
	(7.54)		(5.93)	(3.41)		(2.55)
Family care	0.0710^***^	−0.0351	0.0799^***^	0.119^***^	0.0168	0.137^***^
	(3.01)	(−0.65)	(2.76)	(3.84)	(0.26)	(3.34)
Full-time study	0.0670^***^	0.0822^**^	0.0623^*^	0.0350	0.0455	0.0344
	(2.60)	(2.00)	(1.90)	(1.06)	(0.93)	(0.77)
Long-term sick	−0.0556^*^	−0.127^***^	0.00422	0.340^***^	0.233^***^	0.424^***^
	(−1.91)	(−2.80)	(0.11)	(7.10)	(3.62)	(5.97)
Constant	2.256^***^	2.251^***^	2.229^***^	4.208^***^	4.216^***^	4.199^***^
	(19.80)	(12.66)	(14.97)	(32.45)	(27.76)	(19.88)
*N*	329,282	146,714	182,568	338,628	150,911	187,717
Sargan-Hansen χ^2^(3)	1.189	1.827	6.006	4.372	2.211	5.229
*First-stage*						
F(4, 51,751)	252.03***					
F(4, 23,245)		104.48***				
F(4, 28,513)			145.57***			
F(4, 51,550)				53.71***		
F(4, 23,145)					35.15***	
F(4, 28,412)						22.45***

*t* statistics in parentheses ^*^p < 0.10, ^**^p < 0.05, ^***^p < 0.01

**Table 3:** Instrumental Variable Fixed Effect Panel Estimates with Age Interactions

	General Health All	General Health Male	General Health Female	GHQ12 All	GHQ12 Male	GHQ12 Female
General Health	0.157^***^	0.191^***^	0.133^***^			
	(4.82)	(3.81)	(3.08)			
General health*Age1	−0.0410^***^	− 0.0401^***^	− 0.0416^***^			
	(−7.69)	(−5.25)	(−5.60)			
General health*Age2	−0.0362^***^	− 0.0147	− 0.0535^***^			
	(−4.52)	(−1.22)	(−4.91)			
GHQ12				−0.193^***^	− 0.195^***^	− 0.193^***^
				(−11.47)	(−9.34)	(−7.30)
GHQ12*Age1				0.018^***^	0.020^***^	0.016^***^
				(5.03)	(4.00)	(3.30)
GHQ12*Age2				0.030^***^	0.038^***^	0.026^***^
				(6.37)	(5.51)	(3.87)
Age	0.0219^***^	0.0202^***^	0.0236^***^	0.019^***^	0.018^***^	0.020^***^
	(24.12)	(14.52)	(19.54)	(19.42)	(12.72)	(14.25)
BHPS	0.228^***^	0.198^***^	0.249^***^	0.267^***^	0.221^***^	0.301^***^
	(25.20)	(15.24)	(19.92)	(22.88)	(13.70)	(17.86)
Couple	0.0939^***^	0.0328^**^	0.140^***^	0.054^***^	0.014	0.083^***^
	(9.45)	(2.29)	(10.34)	(4.14)	(0.79)	(4.33)
Higher education	−0.0422^**^	−0.0282	−0.0530^*^	−0.043^*^	−0.014	−0.063^*^
	(−1.98)	(−0.88)	(−1.89)	(−1.72)	(− 0.40)	(−1.86)
Child 0–2	− 0.0518^***^	−0.0717^***^	− 0.0336^***^	−0.057^***^	− 0.078^***^	−0.039^***^
	(−7.12)	(−6.92)	(−3.30)	(−6.11)	(−6.01)	(−2.95)
Child 3–4	−0.0456^***^	−0.0421^***^	− 0.0437^***^	−0.067^***^	− 0.081^***^	−0.054^***^
	(−6.18)	(−3.87)	(−4.36)	(− 7.00)	(−5.87)	(−4.14)
Child 5–11	−0.00634	−0.0146^**^	0.00342	−0.033^***^	− 0.022^***^	−0.039^**^
	(−1.22)	(−1.97)	(0.48)	(−4.80)	(−2.45)	(−3.63)
Child 12–15	−0.0190^***^	−0.0276^***^	− 0.00990	−0.023^***^	− 0.027^***^	−0.019^*^
	(−3.37)	(− 3.30)	(−1.30)	(− 3.20)	(− 2.62)	(−1.86)
Real Family Income	0.0000508^***^	0.0000468^***^	0.0000581^***^	0.0000507^***^	0.0000429^***^	0.0000632^***^
	(28.31)	(21.34)	(18.81)	(23.33)	(16.25)	(16.12)
Self Employed	0.195^***^	0.202^***^	0.189^***^	0.150^***^	0.150^***^	0.142^***^
	(8.05)	(5.33)	(5.84)	(4.67)	(3.22)	(3.12)
Employed	0.225^***^	0.240^***^	0.214^***^	0.189^***^	0.190^***^	0.189^***^
	(10.05)	(6.62)	(7.59)	(6.23)	(4.22)	(4.58)
Unemployed	−0.276^***^	−0.350^***^	−0.197^***^	0.021	−0.045	0.088
	(−11.48)	(−9.24)	(−6.35)	(0.51)	(−0.81)	(1.44)
Retired	0.144^***^	0.129^***^	0.148^***^	0.070^**^	0.013	0.103^***^
	(6.12)	(3.38)	(4.98)	(2.18)	(0.28)	(2.39)
Maternity leave	0.225^***^		0.206^***^	0.126^***^		0.113^**^
	(7.59)		(5.99)	(3.04)		(2.25)
Family care	0.0695^***^	−0.0331	0.0795^***^	0.128^***^	0.027	0.149^***^
	(2.96)	(−0.61)	(2.75)	(3.91)	(0.40)	(3.37)
Full-time study	0.0685^***^	0.0834^**^	0.0644^*^	0.029	0.036	0.031
	(2.66)	(2.04)	(1.96)	(0.85)	(0.71)	(0.66)
Long-term sick	−0.0513^*^	−0.123^***^	0.00871	0.376^***^	0.275^***^	0.461^***^
	(−1.77)	(−2.71)	(0.23)	(7.18)	(3.99)	(5.78)
Constant	2.087^***^	2.110^***^	2.046^***^	4.555^***^	4.562^***^	4.566^***^
	(17.08)	(11.02)	(12.84)	(25.85)	(22.35)	(15.55)
*n*	329,282	146,714	182,568	338,628	150,911	187,717
Sargan-Hansen χ^2^(3)	1.323	1.547	5.456	4.267	2.047	5.062
*First stage*						
F(6, 51,268)	11.12^***^					
	66.73^***^					
	66.46^***^					
F(6, 22,762)		8.27^***^				
		49.63^***^				
		49.54^***^				
F(6, 27,992)			106.4^***^			
			5597.82^***^			
			2366.27^***^			
F(6, 51,550)				59.65^***^		
				3533.35^***^		
				1748.14^***^		
F(6, 23,145)					36.74^***^	
					1492.25^***^	
					654.83^***^	
F(6, 28.412)						27.45^***^
						2073.62^***^
						1100.02^***^

*t* statistics in parentheses ^*^p < 0.10, ^**^p < 0.05, ^***^p < 0.01

In order to further interrogate the relationship between subjective financial situation and health, analysis is conducted across different age groups, by including interaction effects between age groups and the health measures, and for males and females separately. The age groups of 16 to 29; 30 to 64 and above 65 are chosen to best reflect distinct stages of life, in part to reflect World Health Organisation (WHO) guidelines. It is also known that financial circumstances and aspirations vary over the life course. Differences between genders may occur as females are found to exhibit lower financial literacy as well as greater vulnerability financially, so health concerns could exacerbate these issues [37]. Furthermore, males may be less likely to seek help with health-related issues and this might particularly be the case with a persisting traditional male role of financial provider [38, 39].

## Results

Tables 2 and 3 present the regression results for the health effects, and health effects and health-age interaction effects, on subjective financial situation respectively.

Table 2 indicates that general health has a positive significant effect and mental illness (GHQ12) has a negative significant effect on the individual’s perceived financial situation for the total sample. Similar results are found for males and females. The results in Table 3, where health and age group interactions are also included, further support these effects. For the general health variable on its own, for the whole sample, and then for males and females, the positive coefficients show that improved health leads to improvements in subjective financial situation. For the mental illness (GHQ12) variable on its own, for the whole sample and then for males and females, the negative coefficients show that increases in mental illness reduce an individual’s subjective financial situation.

The interaction effects qualify these effects and show that the above effects diminish across age-cohorts. This is because the coefficients for General health*Age1 and General health*Age2 have negative signs for the whole sample, males, and for females. This means that the positive effect of general health on subjective financial situation is reduced across older cohorts compared to the youngest cohort.

In the case of mental illness (GHQ12), the interaction effects for GHQ12*Age1 and for GHQ12*Age2 for the whole sample, males and for females are positive. This means that the effect of having mental illness that reduces subjective financial situation is less for older cohorts compared to the youngest cohort.

## Discussion

Current UK health policy focuses on health as an asset, in the context of integrated care systems and it seeks to promote a more inclusive and productive society, with a focus on the general flourishing of individuals [40, 41]. More general social welfare policy also now promotes the pursuit of well-being of which subjective financial situation is a key component [27].

The literature exploring the relationship between health and financial situation has tended to focus on either the importance of income on health, acknowledging that health can also influence income, and financial burden as a result of out-of-pocket healthcare expenses, restricted participation in the labour market and reduced potential for earnings. The literature has also identified that there are distinct effects of subjective financial well-being on health.

Building on this latter literature, this study focusses on the causal impact of health on subjective financial situation, controlling for the influence of actual income and work status. The advantages of the study are that it draws upon large-scale longitudinal data that facilitated the use of fixed-effects instrumental variable panel-data analysis to identify causal effects. The results show that improvements in general and mental health for both males and females can improve their subjective financial situation. It is also found that the effects change across age groups for both males and females, and that they differ for general health and mental illness. Consequently, these results suggest that linking healthcare to include guidance and advice on the individual’s financial situation particularly across age cohorts, recognising the types of health effects, could enhance well-being and contribute to current health policy objectives [11].

The literature has typically found that older adults exhibit more financial satisfaction than younger adults [42, 43] even at very low levels of income [44]. The above results show that variations in health can qualify these insights. In particular, perhaps through a loss of capability [45], the link between general health and ageing can reduce the positive effect of greater health on the subjective financial situation of individuals. In the case of mental health, however, perhaps through greater resilience, the link between mental illness and reductions in the subjective financial situation of individuals is reduced across ageing cohorts. It is known, for example, that relatively greater social support and links to social organisations in older age groups can counter reductions in mental health through social isolation [46]. It remains that further research is required to identify which health conditions contributing to general health drive the differences compared to mental health to better target policy.

Despite the contributions of the study, the following limitations remain. The synthesised measure of general health has introduced some approximation, which has been partly controlled for by correcting for heteroscedasticity and including a fixed effect to differentiate between the underlying BHPS and USS datasets. Furthermore, due to the long time-series of the data, the availability of instruments was limited and had to be derived from within the data. Although, these instruments passed the diagnostics tests of relevance and validity, further exploration of other instruments could be carried out, for example, acquiring measures of the actual supply-side of healthcare. This is likely to require shorter-time period data as longitudinal data on the availability of healthcare assets is not readily available, but may be extracted, say, from health authority audit data.

## Conclusions

Although the SDH are well known, and there has been research that has examined the impact of subjective financial well-being on health, there has been no research on the reverse impacts of health on the subjective financial situation of individuals, which is linked to their well-being. This is important in the context in which health is viewed as an asset that can have cross-cutting impacts across all domains of individuals’ lives and, moreover, that subjective financial well-being is considered to be an important feature of well-being which is now monitored as part of UK social welfare policy. Health is likely to influence an individual’s well-being based on their feelings of their subjective financial situation regardless of the level of income and employment status. This is because the latter will not fully measure the sufficiency of access to resources to meet an individuals’ needs, their sense of social comparison and expectations connected with differences in income and work status as well as potential future challenges.

This paper has identified that improvements in both general health and reductions in mental illness can improve the subjective financial situation of both males and females. However, in the case of general health the improvement can reduce across ageing cohorts. In the case of mental health, the negative influence of mental illness on subjective financial situation, is reduced across ageing cohorts. This insight provides evidence in favour of linking health care to employment policy and financial advice and support for individuals. However, this needs to be nuanced depending on the health conditions and stage of the life course. The impact of these needs to be further researched.

## Data Availability

The datasets analysed during the current study are available from the UK Data Archive Study Number 6614 https://beta.ukdataservice.ac.uk/datacatalogue/studies/study?id=6614, 10.5255/UKDA-SN-6614-12

## Abbreviations

SDH: Social Determinants of Health
BHPS: British Household Panel Survey
USS: Understanding Society Survey
GHQ12: 12 item General Health Questionnaire
WHO: World Health Organisation
UK: United Kingdom
US: United States
NHS: National Health Service
